# P-1798. Identifying Early Influenza Virus Infections in a Human Infection Model

**DOI:** 10.1093/ofid/ofaf695.1967

**Published:** 2026-01-11

**Authors:** Alana V Brown, Jack G Anderson, Nicholas O’Grady, Clare Camilleri, Sepideh Naderi, Rachel Myers, Edwin Wilbur Woodhouse, Julie M Steinbrink, Thomas W Burke, Christopher W Woods, Micah T McClain

**Affiliations:** Duke University School of Medicine, Durham, North Carolina; Duke University, Durham, North Carolina; Duke University, Durham, North Carolina; Biomeme, Philadelphia, Pennsylvania; Biomeme, Philadelphia, Pennsylvania; Duke University, Durham, North Carolina; Duke University Medical Center, Durham, NC; Duke University Medical Center, Durham, NC; Duke University, Durham, North Carolina; Duke University, Durham, North Carolina; Duke University, Durham, North Carolina

## Abstract

**Background:**

Early, accurate detection of respiratory viral infections is critical for clinical and public health interventions. We previously developed a whole blood mRNA detection assay (HR-B/V) measuring host gene expression biomarkers in circulating leukocytes that accurately discriminates between viral and bacterial acute respiratory infections. Herein, we evaluated performance of these immune biomarkers for detection of viral infection at early timepoints following influenza exposure using controlled human infection models (CHIMs).

**Methods:**

Serial samples from 30 symptomatic, infected subjects across five influenza (H1N1 and H3N2) CHIM studies were analyzed using the Franklin Integrated Sample Prep (ISP), a sample-to-answer real-time (RT) PCR platform with ∼1 hour run-time. Blood samples (from pre-inoculation through resolution of disease) were loaded into ISP cartridges for RNA extraction and multiplex RT-PCR. Cycle threshold values were normalized, and gene expression levels were compared to clinical and virologic outcomes.

**Results:**

Timing of symptom onset after inoculation was variable, but occurred approximately 50 hours after viral exposure, with peak symptoms occurring on average at ∼90 hours. Expression of genes depicting canonical antiviral immune responses was seen as early as 21 hours after inoculation. Most subjects (29/30) exhibited upregulation of ≥ 1 antiviral response gene at or before 36 hours post inoculation, and 73% (22/30) had ≥ 1 antiviral response genes exhibit increased expression prior to symptom onset. A viral model built from these genes correctly diagnosed infection in 29/30 subjects at ≥ 1 acute timepoint.

**Conclusion:**

A host response-based mRNA assay targeting genes involved in canonical antiviral innate immune responses and measured on a rapid, field-deployable platform, shows promise for detection of early, even pre-symptomatic influenza infection.

**Disclosures:**

Clare Camilleri, n/a, Biomeme, Inc.: Employed by Biomeme|Biomeme, Inc.: Stocks/Bonds (Private Company) Sepideh Naderi, n/a, Biomeme Inc: Employee Rachel Myers, PhD, Biomeme: Royalties on patents for gene expression classifiers of fungal infection Julie M. Steinbrink, MD, MHS, Biomeme: patents for gene expression classifiers of fungal infection|McGraw Hill Publishing: royalties Thomas W. Burke, PhD, Biomeme: Advisor/Consultant|Biomeme: Ownership Interest Christopher W. Woods, MD, MPH, Biomeme: Patents on differentiating bacterial from viral infections|Biomeme: Ownership Interest|Biomeme: Stocks/Bonds (Private Company) Micah T. McClain, MD, PhD, Biomeme: patents for gene expression classifiers of fungal infection|Darwin Biosciences: Board Member|UpToDate: Advisor/Consultant

